# The role of polymorphic ERAP1 in autoinflammatory disease

**DOI:** 10.1042/BSR20171503

**Published:** 2018-08-29

**Authors:** Emma Reeves, Edward James

**Affiliations:** 1 Centre for Cancer Immunology, University of Southampton, University Hospital Southampton, Tremona Road, Southampton SO16 6YD, U.K.; 2 Institute for Life sciences, University of Southampton, Southampton SO17 1BJ, U.K.

**Keywords:** Antigen processing and presentation, autoimmunity, ERAP1, inflammation, MHC

## Abstract

Autoimmune and autoinflammatory conditions represent a group of disorders characterized by self-directed tissue damage due to aberrant changes in innate and adaptive immune responses. These disorders possess widely varying clinical phenotypes and etiology; however, they share a number of similarities in genetic associations and environmental influences. Whilst the pathogenic mechanisms of disease remain poorly understood, genome wide association studies (GWAS) have implicated a number of genetic loci that are shared between several autoimmune and autoinflammatory conditions. Association of particular HLA alleles with disease susceptibility represents one of the strongest genetic associations. Furthermore, recent GWAS findings reveal strong associations with single nucleotide polymorphisms in the endoplasmic reticulum aminopeptidase 1 (ERAP1) gene and susceptibility to a number of these HLA-associated conditions. ERAP1 plays a major role in regulating the repertoire of peptides presented on HLA class I alleles at the cell surface, with the presence of single nucleotide polymorphisms in *ERAP1* having a significant impact on peptide processing function and the repertoire of peptides presented. The impact of this dysfunctional peptide generation on CD8+ T-cell responses has been proposed as a mechanism of pathogenesis diseases where HLA and ERAP1 are associated. More recently, studies have highlighted a role for ERAP1 in innate immune-mediated pathways involved in inflammatory responses. Here, we discuss the role of polymorphic ERAP1 in various immune cell functions, and in the context of autoimmune and autoinflammatory disease pathogenesis.

## Introduction

Autoimmune and autoinflammatory diseases are a leading cause of mortality and morbidity worldwide. Although individually these diseases are often regarded as rare in their prevalence, collectively they represent a diverse collection of diseases with the commonality of self-directed tissue damage through innate and adaptive immune responses [1]. Autoimmune diseases are largely categorized based on the involvement of the adaptive immune response, with the presence of autoreactive T cells and antibodies. Additionally, these disorders can be considered as systemic, multiorgan conditions, including systemic lupus erythematosus and rheumatoid arthritis, or organ-specific such as Type 1 diabetes (T1D), multiple sclerosis (MS) and Graves’ disease. At the opposite end of the spectrum are autoinflammatory diseases, which are typically categorized by repeat episodes of fever, rash and arthritis that arise due to defects in the inflammatory response pathways controlled by the innate immune system. Those diseases that are classified toward the autoinflammatory end of the spectrum, such as Crohn’s disease, have a strong IL-23R component, whereas those that are deemed primarily autoimmune in their course of pathogenesis, such as T1D and MS, are not generally associated with IL-23R [2]. Interestingly, a number of conditions display mixed characteristics of both autoimmunity and autoinflammation e.g. Behçet’s disease (BD), inflammatory bowel disease (IBD), ankylosing spondylitis (AS) and psoriasis, where IL-23R may be a factor in disease pathogenesis. Susceptibility to autoimmunity and autoinflammation is influenced by both genetic and environmental factors. The classification of the broad spectrum of autoimmune conditions through to autoinflammatory diseases and their clinical and immunological characteristics is reviewed in [2]. Recently, advances in genetic analysis through genome wide association studies (GWAS) have highlighted several disease-associated loci that are linked with susceptibility to a growing number of autoimmune conditions [3]. These GWAS studies are beginning to highlight shared patterns of genetic associations, such as cytokine and cytokine receptors, inflammatory pathways and proteins involved in T-cell activation. Interestingly, the major histocompatibility complex (MHC) alleles, which function to present peptide antigens to T cells, represent some of the strongest genetic associations with autoimmune and autoinflammatory conditions, such as AS, BD, psoriasis and T1D [4]. Here, we will discuss the contribution of endoplasmic reticulum aminopeptidase 1 (ERAP1), primarily responsible for final peptide trimming in the endoplasmic reticulum (ER) for MHC class I (MHC I) loading, in several autoimmune and inflammatory diseases.

## Biological functions of ERAP1: antigen processing

**Figure 1:**
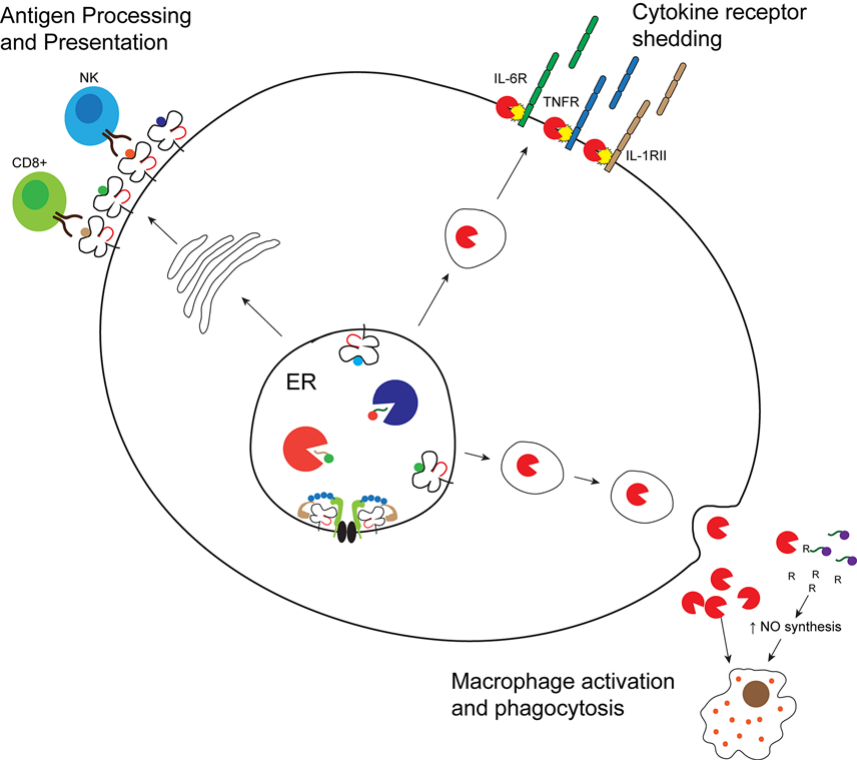
The cellular localization and functions of ERAP1 in autoinflammatory and autoimmune disease ERAP1 and ERAP2 play a key role in processing of peptide antigens for presentation on MHC I molecules at the cells surface. Alternative role for ERAP1 in immune responses is the facilitation of cytokine receptor shedding for IL-6R, TNFR and IL-1RII at the cell surface, and secretion of ERAP1 to enable macrophage activation and phagocytosis.

ERAP1 is an M1 zinc metalloprotease family member, which contains a transmembrane domain and an active site with GAMEN and Zn-binding HEXXH(X)_18_E motifs [5]. The aminopeptidase was first shown to be significant in regulating the peptide repertoire at the cell surface in 2002, where two independent studies investigating human ERAP1 and the mouse homolog, endoplasmic reticulum aminopeptidase associated with antigen processing (ERAAP), revealed that they were responsible for N-terminal trimming of peptide precursors to generate stable peptide MHC I complexes (pMHC I; Figure 1) [6,7]. These and subsequent studies showed that ERAP1 was able to trim both free peptides and peptides bound to MHC I [8–11]. Along with other members of the antigen processing and presentation pathway, such as MHC I, immunoproteasome, TAP and tapasin, ERAP1 is IFN-γ inducible [6]. The peptide regulating function of ERAP1 was most notably highlighted in knockout studies that showed ERAAP knockout cells elicited robust T-cell responses in WT mice when immunized [12]. ERAP1 knockout cells have a significant reduction in pMHC I expression at the cell surface, up to 30% in humans and 70% in mice, depending on the MHC I allele [12–14]. This is further highlighted from analysis of the immunopeptidome presented by ERAP1 knockout cells, which show both quantitative and qualitative differences to normal cells, with fewer peptides presented and those presented are longer in length [12,15,16]. The exact trimming mechanism of ERAP1 has not been fully determined, though two hypotheses have been proposed: (i) the ‘molecular ruler’ hypothesis where ERAP1 trims free peptides to the optimal amino acid length [17,18] and (ii) the ‘MHC I template’ hypothesis where MHC I acts as the template for ERAP1 trimming, stably binding peptides trimmed by ERAP1 when they reach the correct length [11,19]. The crystal structure of ERAP1 reveals a 4-domain protein that is likely to adopt two distinct conformations: open and closed [9,10]. The open conformation is suggested to be the peptide receptive state of ERAP1, able to bind peptide and then close around the peptide into the enzymatically active conformation [10]. Of note, no crystal structures have been solved with a physiological length peptide bound within the active site of ERAP1.

The specificity of ERAP1 reveals an inability to cleave X-proline bonds as well as a distinct hierarchy of trimming activity toward different amino acid substrates with varying properties [11,20]. Indeed, not all peptides require ERAP1 peptide processing activity for MHC I presentation, with a subset of peptides entering the ER at the final optimal length for pMHC I presentation and are detected at the cell surface regardless of ERAP1 expression [12,18]. Conversely, a proportion of peptides that enter the ER are destroyed by ERAP1 activity [12,18,21]. An example of this is the presentation of the CT26 tumor derived cross-protective H2-D^d^ specific antigen, GSW11; in the absence of ERAP1, the level of peptide is increased by ∼75-fold [21]. This highlights the potential role for ERAP1 in disease, with particular emphasis on the modulation of CD8+ T-cell responses in those diseases where these effector functions have a significant role.

**Figure 2:**
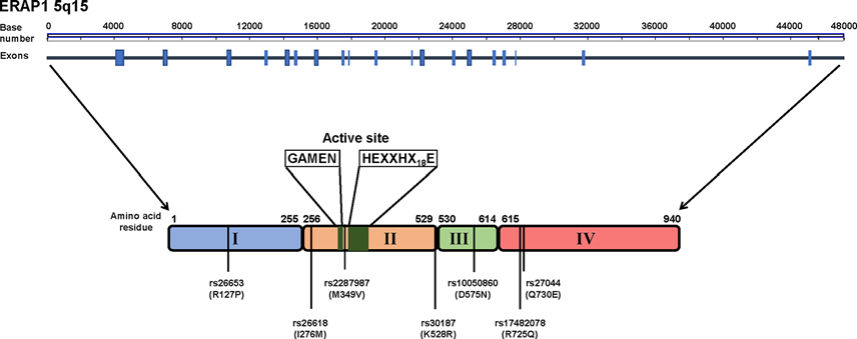
Schematic of ERAP1 The ERAP1 gene spans 48 kb and comprises 19 exons. The ERAP1 protein consists of four domains: domain I in blue, domain II in orange, domain III in green and domain IV in red. The active site position, including GAMEN and HEXXHX_(18)_E motifs, is highlighted in domain II. The locations of the autoinflammatory and autoimmune-associated ERAP1 SNPs, including amino acid variation, are shown.

Single nucleotide polymorphism (SNP) in *ERAP1* are associated with a number of autoimmune/inflammatory conditions, discussed below and reviewed in [22], HPV-induced cancer, hypertension and HCV infection (Table 1, [23–28]). Interestingly, the disease-associated *ERAP1* SNPs do not reside within the active site region of ERAP1 and are located throughout the protein at interdomain junctions or within the regulatory region of domain IV, an area proposed to bind the C-terminal residues of the peptide (Figure 2) [9]. Further analyses of these SNPs and their effect on function revealed significant alterations in peptide trimming enzymatic activity [29]. The most frequently described *ERAP1* SNP, rs30187 encoding the amino acid substitution K528R, showed reduced ability to trim peptides both *in vitro* and *in vivo* [11,24,30,31]. By comparison another reported associated SNP, rs27044 encoding a Q730E substitution was shown to have alterations in both peptide length preferences and trimming specificity [11,31,32]. Interestingly, the effect of individual SNPs on ERAP1 trimming can be combined. Examination of the SNPs K528R and D575N (rs10050860) revealed a hierarchy of ERAP1 trimming activity for different combinations, with the greatest activity demonstrated with an ERAP1 containing K528/N575 and the lowest with R528/D575, suggesting a cumulative effect of SNPs [33]. The highly polymorphic nature of *ERAP1* and the presence of discrete haplotypes consisting of a combination of SNPs were further confirmed in our study of a small cohort of individuals. This study revealed 13 different haplotypes, encoding distinct ERAP1 allotypes with altered function. These allotypes were shown to belong to one of three functional categories: efficient, hypoactive or hyperactive, based on their ability to generate the final model epitope SIINFEHL from its precursor. The most common *ERAP1* SNP combination was found to encode an allotype with five variants, which have been individually associated with a number of diseases (ERAP1 V349, R528, N575, Q725, E730, designated ERAP1*001 and Hap10) and is found in 26.2% of the European Caucasian population [14,34]. Interestingly, this variant encodes a functionally inactive ERAP1 protein [11]. To add to the complexity, ERAP1 is co-dominantly expressed and we showed that both allotypes contribute to the overall ERAP1 trimming function in individuals and its relevance to disease [11,14]. In addition, one study has suggested that the presence of SNPs in *ERAP1* affects both mRNA and protein expression in the cell, with *ERAP1* containing SNPs ‘susceptible’ for spondylarthritis (Table 1) having a greater level of overall expression and enzymatic activity [35]. Due to increasing reports of ERAP1 SNP association with autoimmune/inflammatory conditions, some of which have a strong genetic link with specific HLA alleles, elucidating the contribution of these SNPs on ERAP1 function will prove vital for underpinning the role of ERAP1 in disease pathogenesis.

**Table 1:** Summary of disease-associated *ERAP1* SNPs and imputed *ERAP1* haplotypes encoding amino acid changes

Disease	HLA association	*ERAP1* SNP association	Associated *ERAP* haplotype
Ankylosing spondylitis [28,30]	HLA-B*27 Susceptible alleles: HLA-B*27:02 and HLA-B*27:05 Protective alleles: HLA-B*27:06 and HLA-B*27:09	rs26653 (R127P) rs2287987 (M349V) rs30187 (K528R) rs10050860 (D575N) rs17482078 (R725Q) rs27044 (Q730E)	Susceptible *ERAP1*: M349/K528/D575/R725/Q730 Protective *ERAP1*: V349/R528/N575/Q725/ E730
Behçet’s disease [27,66,67]	HLA-B*51	rs10050860 (D575N) rs17482078 (R725Q)	ERAP1*001/Hap10: P127/M349/R528/N575/Q725/E730
Type 1 diabetes [76]	HLA-DR3, HLA-DR4, HLA-DQ2 HLA-DQ8 HLA-A*02:01 HLA-A*24:02	rs30187 (K528R)	
Psoriasis [23,81–83]	HLA-C*06:02	rs26653 (R127P) rs30187 (K528R) rs27044 (Q730E)	Susceptible *ERAP1*: M349/K528/Q730 +*ERAP2* expression Protective *ERAP1***:** M349/R528/E730 -*ERAP2* expression
Multiple sclerosis [86]	HLA-DR15 Protective allele: HLA-C*05	rs30187 (K528R)	
Inflammatory bowel disease [88]	HLA-C*07	rs30187 (K528R)	
Birdshot chorioretinopathy	HLA-A*29:02	rs10044354 (*ERAP2*)	*ERAP2* expression

## Biological functions of ERAP1: innate immunomodulation

There is an emerging role for ERAP1 in extracellular functions of immune regulation. Whilst these have not yet been extensively studied, there is clear evidence that the antigen processing role of ERAP1 for MHC I presentation is not the sole function of ERAP1. Despite the lack of an obvious ER-retention signal, ERAP1 localizes within the ER. However, additional studies have suggested that ERAP1 can be localized in the cytosol, at the cell membrane as a type II integral membrane protein and secreted from the cell [7,36–38]. It is therefore plausible that ERAP1 may alter its subcellular localization depending on (i) the type of cell expressing ERAP1 and (ii) the environmental changes and stressors exerted in the cell (e.g. in response to inflammatory signals).

Macrophages control and regulate inflammation and responses to invading pathogens through induction of proinflammatory cytokines. Secretion of enzymatically active ERAP1 has been reported in response to LPS and IFN-γ by the macrophage cell line RAW246.7. The secretion of ERAP1 relied upon the induction of IFN-β and TNF-α through TLR-mediated signaling [37]. In addition, the resulting increased intracellular Ca^2+^ levels from cytokine induction led to calmodulin-mediated secretion of ERAP1 [39]. The function of this secreted ERAP1 was shown to enhance/up-regulate macrophage phagocytosis through TLR4 (LPS receptor) signal transduction [37]. Interestingly, when macrophages were treated with enzymatically inactive ERAP1 (containing E354Q) or ERAP1 with altered substrate specificity (containing Q181D), the enhancement of phagocytosis was minimal compared with WT ERAP1 suggesting ERAP1 is required to generate substrates that induce phagocytosis activity. However, as yet, the identity of these phagocytosis inducing substrates are unknown [37]. Further investigation also identified that extracellular-secreted ERAP1 mediates trimming of peptides with N-terminal arginine (Arg) residues, thereby generating free-Arg supply that up-regulates nitric oxide (NO) synthesis [40]. Maximum synthesis of NO is required for blood pressure regulation, through mediation of endothelial cell relaxation, as well as modulating inflammatory responses by specific immune cells such as macrophages [41,42]. Supplies of free-Arg are essential for the maximum synthesis of NO, therefore, the secretion of ERAP1 under inflammatory stimuli could have direct and indirect effects on blood pressure control and disease-associated inflammation via NO-mediated pathways. Interestingly, blood pressure regulation has previously revealed association with ERAP1 activity due to its function in cleaving bioactive peptide hormones angiotensin II and kallidin into angiotensin III and bradykinin respectively [24].

The innate branch of the immune system is the first line of defense for pathogen invasion, relying on pattern recognition receptors to initiate signaling pathways that drive the proinflammatory responses, including the secretion of various cytokines. Upon stimulation with *Eimeria tenella* derived antigen or Adenovirus vectors, the absence of *ERAP1* expression in mice results in increased production of proinflammatory cytokines IL-6, MCP-1, TNF-α and IL-12 as well as an increase in activated and mature NK cells, which correlated with IFN-γ production in those cells lacking ERAP1 [43]. Interestingly, innate immunostimulatory pathways were activated in human PBMCs when exposed to autoimmune-associated ERAP1 variants K528R, Q730E and to a lesser extent K528R/Q730E in comparison with WT ERAP1 [44]. In line with other studies, secreted ERAP1 possesses enzymatic trimming function, and those ERAP1 variants known to have reduced trimming function resulted in higher levels of proinflammatory cytokines IL-1β, IL-6 and TNF-α as well as increased activation of NK and T cells. In particular, K528R potently induced IL-1β and the caspase I/NLRP3 inflammasome pathway [44]. These findings further demonstrate the role of polymorphic ERAP1 in both innate and adaptive immunostimulatory pathways (Figure 1), promoting inflammation via alternative pathogenic mechanisms in addition to the well-characterized antigen processing function of ERAP1.

## Biological functions of ERAP1: cytokine receptor shedding

Cytokines regulate several biological processes such as inflammation, cytotoxicity, antiviral responses and transcriptional regulation of genes. Induction of the biological functions of cytokines is often mediated through binding to distinct cell surface receptors, which activate specific signaling cascades within the cell. ERAP1 has been suggested to function in the shedding of cytokine receptors IL-6R, TNFR1 and IL1RII, known as aminopeptidase regulator TNFR1 shedding in this context (Figure 1) [36,45,46]. IL-6, TNF-α and IL-1 have all been implicated in the inflammatory response and diseases such as AS, MS and IBD, therefore highlighting a possible link between ERAP1 and inflammation.

ERAP1 is a type II integral membrane protein with a transmembrane domain region, however does not possess ectodomain cleavage activity [36]. As the transmembrane domain region of ERAP1 overlaps with the signal peptide sequence, ERAP1 may exist as both a transmembrane and soluble protein in cells. An association between TNFR1 and ERAP1 was identified through a yeast-2-hybrid screening, revealing ERAP1 could bind to the receptor, but not cleave the ectodomain region [36]. This suggested a role of ERAP1 in acting as a binding partner to form a complex with another protein containing ectodomain cleavage activity, thereby ‘facilitating’ the receptor shedding via an indirect mechanism. This idea was further supported by the identification of nucleobindin-2 and RNA-binding motif gene, X chromosome that form complexes with ERAP1 and promotes an increase in TNFR1 cleavage activity [47,48]. Interestingly, an intact catalytic domain of ERAP1 was shown to be required for IL-6R shedding but not TNFR1 shedding, with ERAP1 able to bind to both soluble and membrane bound IL-6R [36,46]. It is interesting to speculate that ERAP1 regulates the biological function of cytokines through receptor shedding. However, there have been no further investigations to support and confirm this role and so the mechanism of action of ERAP1 in cytokine receptor shedding remains elusive. It is tempting to speculate that polymorphic variants of ERAP1 with altered functional activity may alter the level of cytokine receptor shedding as a result of reduced or overactive activity, as well as altering the ability to form the complexes essential for the promotion of cytokine receptor cleavage. This would alter the levels of cytokine receptors at the cell surface and in serum and therefore the signaling cascades mediated by cytokine receptor resulting in the promotion of a proinflammatory environment and the induction of autoinflammation/immunity.

## Biological functions: ERAP2

In humans, a second ER resident aminopeptidase, ERAP2, exists and shares 49% homology with ERAP1 with most sequence identity spanning the active site consensus region conserved in M1 metalloprotease members [5]. The involvement of ERAP2 in antigen processing has not been as extensively characterized as ERAP1, and as the tissue distribution of ERAP2 is noted to be different to both ERAP1 and MHC I expression, the involvement and contribution of ERAP2 in this pathway is not as obvious as ERAP1. When comparing peptide trimming functions, ERAP2 confers altered substrate handling and amino acid preferences compared with ERAP1. Interestingly, ERAP2 preferentially cleaves basic amino acids, and shows particular preference for Arg, and cleavage activity dramatically decreases with substrates longer than 8/9amino acids [49]. ERAP1 and ERAP2 can form heterodimers that generate final peptides from certain precursor extensions (amino acid sequence specific) at a faster rate *in vitro* than their individual counterparts [50,51]. However, since these heterodimers only account for ~30% of ERAP1 and ERAP2 in cells, the biological significance of this *in vivo* has not been determined.

ERAP2 has little polymorphic variation compared with ERAP1. The most widely described SNP in ERAP2, Lys392Asn, (rs2549782) shows altered enzymatic function and substrate specificity of ERAP2 containing Asn392, with the ability to cleave hydrophobic amino acid residues faster than Lys392 [52]. However, the Asn392 ERAP2 allele is rarely expressed in individuals due to the high linkage disequilibrium with the SNP responsible for lack of ERAP2 expression (rs2248374). Approximately 25% of individuals lack expression of ERAP2 as a result of alternative splicing to generate a truncated form of ERAP2, which undergoes degradation through nonsense-mediated decay of mRNA [53].

## Polymorphic ERAP1-associated autoimmune and inflammatory conditions

### Ankylosing spondylitis

AS is a chronic autoinflammatory condition that belongs to the group of spondyloarthropathies (SpA), comprising several immune-mediated disorders including psoriatic arthritis, reactive arthritis and arthritis associated with IBD. AS is a systemic disease affecting the sacroiliac joints of the lower back, however is known to cause other inflammatory features such as acute anterior uveitis (AAU) and IBD. The genetic link between AS and HLA-B*27 has been known for many years, with subsequent evidence revealing susceptibility with HLA-B*27:02 and HLA-B*27:05 subtypes but protection with HLA-B*27:06 and HLA-B*27:09 alleles [54,55]. In 2007, the presence of non-MHC genetic susceptibility factors linked with AS confirmed the involvement of other genetic factors that contribute to disease. SNPs in *ERAP*1 (*P* = 1 × 10^−26^) and *IL-23R* were significantly linked to AS disease susceptibility, conferring 26% and 9% risk respectively [28]. Further studies have also identified a link between protection to AS and the loss of *ERAP2* expression in both HLA-B*27 positive and HLA-B*27 negative AS cases [56]. The link between *ERAP1* SNP and AS has been extensively investigated and is now widely accepted, being replicated in multiple independent studies and ethnic populations (reviewed in [34]).

Initial studies focused on five *ERAP1* SNPs, rs2287987, rs30187, rs10050860, rs17482078 and rs27044, encoding amino acid substitutions M349V, K528R, D575N, R725Q and Q730E that were identified in the original GWAS studies (Figure 1) [28]. A later GWAS study revealed 33 sequence SNPs, including several new *ERAP1* SNP associations with AS, including rs27434 (amino acid position 356) [57]. Interestingly, the WTCCC revealed that the link between *ERAP1* and AS was only observed in individuals with HLA-B*27, a phenomenon that is becoming increasingly evident in other autoimmune conditions that link specific HLA and ERAP1 to disease risk [30,58]. Other risk genes and loci, such as IL-23R SNPs, do not show this distinction between HLA-B*27 positive and negative individuals. When determining the effect of the ERAP1 risk alleles in individuals with AS and AAU compared with AS cases without AAU, the rs30187 variant showed a greater effect on AS and AAU than on AS alone [59]. Interestingly, further investigation has shown the association of *ERAP1* rs30187 and rs27044 with HLA-B*27 positive SpA [60]. The disease-associated SNPs for AS and other autoimmune and inflammatory conditions, including *ERAP1* SNP haplotypes, are summarized in Table 1 and Figure 2.

The most recent investigations of *ERAP1* SNPs have focused on the combination of *ERAP1* SNPs within each chromosomal copy. These highlighted the highly polymorphic nature of *ERAP*1 and that multiple SNPs can occur in distinct combinations. We have demonstrated that those ERAP1 allotype combinations found in AS cases had significantly different peptide processing function than those found in controls. This in turn affected the peptides generated for pMHC I expression and altered the levels of HLA-B*27 expression at the cell surface [14]. Additionally, reduction in ERAP1 expression influences the generation of HLA-B*27 binding peptides by increasing both peptide length and expression of intracellular free heavy chains, impacting on the stability of HLA-B*27 both intracellularly and at the cell surface [58,61]. The HLA-B*27 peptidome was significantly altered in cells containing the five most common AS-associated SNPs, generating suboptimal ligands for HLA-B*27 presentation in comparison with those generated with WT ERAP1. Importantly, ERAP1 containing AS-associated ‘susceptible’ SNPs (Table 1) was shown to rapidly degrade HLA-B*27 epitopes, resulting in a global reduction in levels of HLA-B*27 at the cell surface [62].

Taken together, these data suggest a significant role for ERAP1 in generating the optimal peptidome for stable HLA-B*27 expression. Since HLA-B*27 has a high tendency to misfold and form homodimers, due to an unpaired cysteine residue at position 67, either at the cell surface or intracellularly, the generation of suboptimal peptides by dysfunctional ERAP1 variants seems a likely link to AS disease pathogenesis. Intracellular misfolding of HLA-B*27 with suboptimal peptide cargo would likely activate the unfolded protein response (UPR), a mechanism to maintain homeostatic ER environment and combat ER stress. The activation of this pathway has been shown to result in an enhancement of proinflammatory cytokine release, including IL-23. Furthermore, there is increasing evidence that UPR may be up-regulated in cells expressing HLA-B*27 in response to misfolding and protein aggregation [63]. Conversely, at the cell surface HLA-B*27 present to both CD8+ T cells and NK cells, with HLA-B*27 recognized by KIR3DL1 receptor on NK cells [64]. The formation of homodimers at the cell surface may be facilitated by loading of unstable and suboptimal peptides that have a rapid dissociation rate, implicating the ERAP1 variants with dysfunctional activity on activation or inhibition of KIR receptors through HLA-B*27 ligand generation. Interestingly, KIR3DL1 and KIR3DL2 recognize cell surface homodimers, with KIR3DL2 levels found to be significantly up-regulated in HLA-B*27 positive AS cases [65]. This suggests a downstream role for ERAP1 in the activation and inhibition of T cell and NK cell responses.

## Behçet’s disease

BD is a multisystemic autoinflammatory disorder that presents with ocular and skin inflammation, orogenital ulcers and often arthritis and inflammation of the bowel. The prevalence of BD is most common in Turkey and Japan, as well as in the countries of the ‘Silk Road’. The strongest risk factor for susceptibility to BD is the presence of HLA-B*51 allele. Other genetic associations include IL-10 and IL-23R, which are notably also implicated in Crohn’s disease (CD), ulcerative colitis (UC) and AS. *ERAP1* SNPs, rs10050860 and rs17482078 encoding D575N and R725Q variants respectively (Figure 2), are in strong linkage disequilibrium and are associated in patients with BD with uveitis [66,67]. This contrasts with AS, where these two *ERAP1* SNPs were demonstrated to be protective against disease susceptibility (Table 1) [30]. Furthermore, ERAP1 is in epistasis with HLA-B*51 positive BD in Spanish and Turkish populations [66,68]. Further investigation into the role of *ERAP1* SNPs in BD revealed a single ERAP1 protein allotype, designated ERAP1*001 (reported as Hap10), had an 11-fold increase in risk of BD in those individuals homozygote for ERAP1*001 and positive for HLA-B*51 [27]. ERAP1*001 contains five SNPs frequently reported with autoimmune disease association (ERAP1 M349V, K528R, D575N, R725Q and Q730E) and further confirms that ERAP1 SNPs occur in multiple combinations as previously reported [14,28,34]. ERAP1*001 has reduced trimming activity, previously shown to alter the generation of final peptide epitopes, and was proposed to significantly affect the peptide repertoire on HLA-B*51 alleles [11,34]. HLA-B*51 peptidome analysis revealed peptide-binding motif to contain Ala or Pro residues at p2 and C-terminal Val or Ile residues, which constitute two subpeptidomes, with peptides containing p2 Pro having a higher binding affinity to HLA-B*51. *ERAP1* containing SNPs R127P, I276M (rs26618), K528R and Q730E affects the peptidome by destroying peptides with p2 Ala residue unless the p1 amino acid was resistant to ERAP1 trimming [69]. Furthermore, the BD-associated low activity variant ERAP1*001 revealed generation of longer length peptides, an increase in the subpeptidome with p2 Pro peptides, and a peptidome with overall lower affinity for HLA-B*51 [70]. This provides clear evidence for disease-associated ERAP1 allotype on altering the peptide repertoire in HLA-B*51 individuals with ERAP1*001 allotype. This is likely to significantly impact the magnitude and specificity of T-cell responses, although this is yet to be confirmed.

## Birdshot chorioretinopathy

Birdshot chorioretinopathy (BSCR) is a rare ocular autoinflammatory disorder that has a strong genetic association with the HLA-A*29:02 allele. BSCR manifests itself as a progressive chronic posterior uveitis that eventually results in retinal atrophy and blindness. The frequency of HLA-A*29:02 is approximately 7% of healthy individuals, but is present in >95% BSCR cases, and the disease is most often reported in middle-age to elderly Europeans. GWAS studies conducted in a Dutch and Spanish cohort revealed a significant association between the *ERAP2* rs10044354 SNP and BSCR (Table 1). Further expression analysis highlighted this SNP to be responsible for altered *ERAP2* mRNA and protein expression, concluding that individuals that were either heterozygous or homozygous for the risk allele rs10044354 (T) had a higher mRNA and protein expression, correlating with risk of BSCR [71]. Although most HLA-associated autoimmune conditions have shown strong linkage with ERAP1, BSCR and the linkage with ERAP2 expression may highlight a role for ERAP2 in controlling the generation or destruction of specific epitopes for HLA-A*29:02. Due to the highly polymorphic nature of *ERAP1* within the population and the likelihood that HLA-A*29:02 individuals with BSCR will express ERAP1 variants, the HLA-A*29:02 peptidome was first analyzed in the context of ERAP1 variants with the lack of ERAP2 expression. Interestingly, the effect of ERAP1 variation on peptide presentation by HLA-A*29:02 revealed that ERAP1 with high trimming activity resulted in a peptidome consisting largely of 9-mer peptides with bulkier side chains, and an overall higher affinity of HLA-A29:02 ligands than the less active ERAP1 variants [72]. In a more recent study, to determine the direct contribution of ERAP2 on the HLA-A*29:02 peptidome, ERAP2 was revealed to have a quantitative effect on the peptides presented. Although there were few alterations in global affinity of HLA-A*29:02 ligands in the presence and absence of ERAP2, a significant number of peptides were >9 amino acids in length and contained a hydrophobic p1 residue in the presence of ERAP2 [73]. These peptidome analysis studies suggest a role for ERAP2 in the protection of longer peptides from destruction by ERAP1 activity, as well as the destruction of those ligands containing ERAP2 susceptible p1 residues, which are unfavored by HLA-A*29:02. The organ specific nature of this condition may suggest a role for ocular-specific antigens that result in a high T-cell infiltrate and CD8+ antigen-specific T-cells responses in the eye. Three candidate antigens derived from the retina-specific S-antigen have previously been proposed, with one S-antigen peptide, the 9-mer epitope VTLGILVSY, containing an ERAP2 susceptible Arg N-terminal extension [74]. This antigen is a candidate for HLA-A*29:02 binding and may prove to be an immunogenic antigen that is capable of activating CD8+ T cells in the presence of ERAP2 expression. In addition, the role of NK cells and KIR receptors in the pathogenesis of BSCR cannot be ruled out, as the potential ERAP2-specific alterations on the peptidome of HLA-A*29:02 may affect the interaction between HLA and NK KIR receptors. Further studies to highlight potential pathogenic ligands, and the resulting effect of ERAP2 expression on the subsequent inflammatory response, either by CD8+ T cell or NK cell activation, will be essential for determining the contribution of ERAP2 to BSCR.

## Type 1 diabetes

The pathogenesis of the autoimmune disorder, T1D, is attributable to several genetic factors and environmental influences and is characterized by the destruction of insulin producing β-cells of the pancreas. Whilst being regarded as organ-specific, the subsequent metabolic abnormalities of T1D can lead to a number of long-term complications such as kidney failure, ischemic heart disease and stroke. As well as a strong genetic association with HLA II alleles, in particular HLA-DR3, -DR4, -DQ2 and -DQ8, and CD4+ T-cell expansion, there is emerging evidence for a role of CD8+ T cells in β-cell death [75]. In 2008, an association between the *ERAP1* rs30187 (K528R) SNP and T1D susceptibility was reported, with a significance of *P*=0.003895 (Table 1) [76]. In the context of antigen processing and HLA-linkage, β-cell destruction is associated with the presentation of specific epitopes derived from the signal peptide region of the pre-proinsulin protein (PPI) presented on HLA-A*02:01 and HLA-A*24:02 alleles [75,77]. Furthermore, the processing of these PPI epitopes was shown to be dependent on ERAP1 activity [77]. This study highlighted two possible pathways for PPI processing and HLA presentation, those that are released into the cytosol and require TAP for transport into the ER, and those that are cleaved and released directly into the ER lumen. However, both processes are likely to require processing of PPI precursors by ERAP1 [77] and suggest a role for altered function of ERAP1 in dysfunctional presentation of these autoreactive PPI epitopes to CD8+ T cells.

## Psoriasis

Psoriasis is a chronic immune-mediated inflammatory disorder affecting the skin and is characterized by hyperproliferation and differentiation of keratinocytes. Like other autoimmune conditions, the strongest genetic association with psoriasis is with a specific HLA allele HLA-C*06:02, with those presenting with early onset (or type 1 psoriasis) having a higher prevalence of HLA-C*06 expression [78,79]. Interestingly, there is a high level of phenotypic variability that may result from the differing disease genetic risk factors, including the presence or absence of HLA-C*06, as well as a higher degree of heritability reported in those individuals with early onset disease (<40 years [78,79]). Similarly to AS and BD, further investigation revealed significant evidence for the interaction between ERAP1 and HLA-C loci in psoriasis, with a combined significance of *P* = 6.95 × 10^−6^ [23]. The specific role of *ERAP1* SNPs was highlighted by the association of rs27524 in HLA-Cw6 positive individuals [23]. Interestingly, *IL-23R* rs11209026 and *ERAP1* rs27524 (intronic) were associated with pediatric onset psoriasis (<18 years) in HLA-C*06 positive individuals [80], whilst additional investigation revealed the association of another *ERAP1* SNP, rs26653 (R127P *P=*0.00006), which was confined to disease onset after puberty (10–20 years), but is not dependent on HLA-C*06 expression [81]. More recent investigation into the classification of early onset psoriasis has confirmed the association of *ERAP1* rs30187 and rs27044 (K528R and Q730E) only in HLA-C*06 positive individuals. It also revealed a significant association of an ERAP1 haplotype based on minor alleles at these SNP positions (*P* = 5.2 × 10^−3^), as well as a haplotype that increased disease susceptibility, containing expression of *ERAP2* along with *ERAP1* containing rs2287987, rs30187 and rs27044 SNPs (M349, K528 and Q730, Figure 2). This compared with a haplotype that reduced disease risk, which had no ERAP2 expression along with ERAP1 M349, R528 and E730 (Table 1) [82,83]. The enzymatic activity of this ERAP1 haplotype has been shown to be efficient and therefore suggests a role in aberrant peptide generation for HLA-C*06 stable peptide expression at the cell surface.

## Multiple sclerosis

 MS is an autoimmune neurological condition, affecting the central nervous system that results in inflammation and demyelination of the nerves. Like many other autoimmune conditions, genetic and environmental factors are likely to play a role in pathogenesis, along with suggestions that viral infections may also be implicated [84]. The strongest genetic risk factor for MS is the MHC II HLA-DR15 haplotype [85]. Interestingly, the HLA-C*05 alleles was demonstrated to have a protective association with MS; however, the functional *ERAP1* variant rs30187 (K528R) has been associated with MS disease susceptibility (Table 1) [86,87]. Furthermore, as ERAP1 is not known to function in the processing of antigens for MHC II presentation, the role of ERAP1 in MS susceptibility may be a result of its alternative cellular functions such as cytokine receptor shedding and mediating cytokine production, with TNFR1 highlighted as a susceptibility locus for MS [86].

## Inflammatory bowel disease

IBD encompasses a number of conditions that involve relapsing and remitting chronic inflammation of the bowel and small intestine, and include UC and CD. The functional variant rs30187 (K528R) ERAP1 confers disease susceptibility to CD and IBD [86]. More recently, there has been reports of an association between ERAP1 K528R and the expression of HLA-C*07 allele in patients with IBD (Table 1) [88]. Interestingly, AS and CD have a close clinical relationship, with approximately 10% of individuals with AS presenting with IBD [89]. The K528R associated with CD has reduced enzymatic activity and is therefore likely to alter the peptide repertoire presented on HLA-C*07 alleles. The consequence of this in disease pathogenesis may be the modified interaction of KIR receptors with the pMHC I complex at the cell surface. Several HLA-C alleles are known to be ligands for KIR receptors and are important in NK cell target recognition through these interactions [90]. Interestingly, there is evidence to suggest that KIR2DL2 and KIR2DL3 interaction with HLA-C may confer susceptibility to CD [91], so it is tempting to speculate the involvement of ERAP1 variant K528R in generating aberrant peptides results in suboptimal pMHC I complexes, which ultimately alter the NK cell-targeted response.

*ERAP1* SNPs have also been investigated in Familial Mediterranean fever (FMF), an autosomal recessive autoinflammatory disorder resulting in fever, skin rash and inflammation of the peritoneum. When examined in a pilot study, the number of *ERAP1* SNPs found in patients with FMF was higher than those with UC [92]. Due to the lack of a known HLA association with FMF, this may suggest a tentative link and an alternative role of ERAP1 variants in the initiation of inflammation through innate immune-mediated pathways.

## Conclusions

Many autoimmune conditions confer strong HLA genetic components to the susceptibility of disease; however, the presence of these specific HLA alleles alone is not sufficient to cause disease. Interestingly, the diseases largely associated with MHC I are largely innate immune mediated and fall into the autoinflammatory end of the spectrum, whereas those that convey a strong MHC II association are most associated with the adaptive immune response [2]. In addition, those diseases discussed here that show mixed characteristics of both autoimmunity and autoinflammation, such as AS and psoriasis, have a strong MHC I association. Advances in GWAS studies in the last decade have highlighted several key regulators and pathways implicated in the pathogenesis of multiple autoimmune and inflammatory diseases.

The epistatic interaction of *ERAP1* SNPs with specific HLA in many of these diseases suggests a key role for peptide generation and cell surface presentation as a significant factor of disease pathogenesis. CD8+ T cell and NK cell activation to specific peptide epitopes at the cell surface is determined by MHC I biology. Binding of optimal peptide cargo in the ER promotes the stable MHC I folding and complex formation for export and presentation at the cell surface. The binding of stable, final length peptide epitopes with correct peptide sequence, including optimal amino acid anchors unique to each HLA molecule, influences both the half-life and the stability at the cell surface. Peptides are known to fall into three categories for their requirement on ERAP1 trimming function: independent, dependent and sensitive [12,13]. ERAP1 allotypic variants have altered ability to trim the peptide epitope precursors to the optimal length and therefore may significantly change the presented peptidome, influencing the abundance of certain peptides in the context of highly active or inactive ERAP1 and the presence or absence of ERAP2 expression [16,49,72]. In addition, changes in ERAP1/ERAP2 expression may destroy potentially disease-protective epitopes and/or increase the generation of disease-susceptible epitopes. Interestingly, pathogenic epitopes have been proposed as a potential disease mechanism in AS, termed ‘arthritogenic peptide hypothesis’, and is an attractive proposal considering the altered ERAP1 function of ‘susceptible’ and ‘protective’ ERAP1 variants [93]. However, to date, studies have been unsuccessful in identifying a disease-specific epitope. Conversely, a similar hypothesis is proposed in BSCR, where the disease is organ-specific and limited to inflammation of the eye. Several pathogenic epitopes have been predicted from the retinal specific S-antigen, with one epitope containing the precursor optimal for ERAP2 trimming activity (R-VTLGILVSY), suggesting a role for the expression of ERAP2 in patients with BSCR [74].

Biological characteristics of specific MHC I demonstrate the importance for optimal peptide cargo in stable MHC I presentation. HLA-B*27 has unusual folding characteristics and has the propensity to form homodimers both intracellularly and at the cell surface, likely as a result of unstable peptide loading [94,95]. The BD-associated MHC I, HLA*B51 also displays unusual characteristics, with slow assembly, inefficient folding and a dependency on tapasin, suggesting a requirement for optimal peptide editing and loading [96]. The effects of dysfunctional ERAP1 activity may therefore exacerbate these characteristics through aberrant peptide generation, resulting in NK cell-mediated lysis [97]. Functional variations of ERAP1 are deemed protective in some conditions and susceptible in others, for example D575N and R725Q in BD versus AS respectively [30,66]. Investigations into the impact of these ERAP1 variants show significant alterations in the quality and quantity of peptides displayed at the cells surface, likely in turn to affect T cell and NK-mediated inflammatory responses. Peptidome analysis studies are starting to uncover the effect of ERAP1 SNPs and in turn their altered functional properties on the nature of the peptides presented; however, further extensive analysis for alterations in individual peptides is required. Further investigation to identify similarities and differences between the effect of ERAP1 on the peptidome may prove essential for identifying ERAP1 and the peptidome as a therapeutic target for these diseases. Interestingly, small molecular modulators of ERAP1 and ERAP2 function may act to alter resulting immune responses [98]. Interestingly, DG013A, a first generation pseudopeptide containing a phosphinic group was a potent inhibitor of ERAP1 and ERAP2 activity and was demonstrated to enhance CD8+ T-cell responses against a murine colon carcinoma cell line [99]. These encouraging results, in the context of cancer, have shown that reducing ERAP1/ERAP2 activity can alter the immune response, which may well prove protective in AS and BSCR. Alternatively, activating ERAP1 may prove useful in diseases such as BD, where ERAP1 associated with disease has reduced function.

Recently, however, studies have begun to investigate an alternative role for ERAP1 in processes such as macrophage activation and phagocytosis, NK cell activation/inhibition, cytokine receptor shedding and regulation of cytokine secretion. These innate immune regulatory pathways have been shown to be significant in an inflammatory situation, and therefore may prove to be as vital as antigen presentation in the initiation of immune responses. These ‘nonclassical’ roles of ERAP1 in immune regulation highlight the potential function of ERAP1 in both innate and adaptive responses applicable to all diseases associated with ERAP1. Further investigation of these ‘nonclassical’ roles of ERAP1 to elucidate the impact of functional variants within these processes is required and may prove productive in understanding the contribution of ERAP1 to autoimmune and autoinflammatory diseases, particularly in those pathological conditions where HLA is not a strong genetic component.

## Abbreviations

AAU: acute anterior uveitis
AS: ankylosing spondylitis
BD: Behçet’s disease
BSCR: birdshot chorioretinopathy
CD: Crohn’s disease
ERAAP: endoplasmic reticulum aminopeptidase associated with antigen processing
ERAP: endoplasmic reticulum aminopeptidas
FMF: Familial Mediterranean fever
GWAS: genome wide association studies
HLA: human leukocyte antigen
IBD: inflammatory bowel disease
IFN-γ: interferon gamma
IFN-β: interferon beta
IL-1RII: interleukin 1 receptor II
IL-6R: interleukin 6 receptor
LPS: lipopolysaccharide
MHC: major histocompatibility complex
PBMC: peripheral blood mononuclear cells
PPI: pre-proinsulin protein
SNP: single nucleotide polymorphisms
SpA: spondyloarthropathies
T1D: Type 1 diabetes
TNF-α: tumor necrosis factor alpha
TNFR: tumor necrosis factor receptor
UC: ulcerative colitis
UPR: unfolded protein response
